# Supplementary material for: Probe set selection for targeted spatial transcriptomics
Source: Nat Methods. 2024 Nov 18;21(12):2260–70. doi: 10.1038/s41592-024-02496-z (PMC11621025; doi:10.1038/s41592-024-02496-z)
Supplement: Supplementary file 1 — Supplementary Figs. 1–7 and Note 1. [file 41592_2024_2496_MOESM1_ESM.pdf]

nature methods

Supplementary information https://doi.org/10.1038/s41592-024-02496-z

Probe set selection for targeted spatial transcriptomics

In the format provided by the authors and unedited

Supplementary Figures

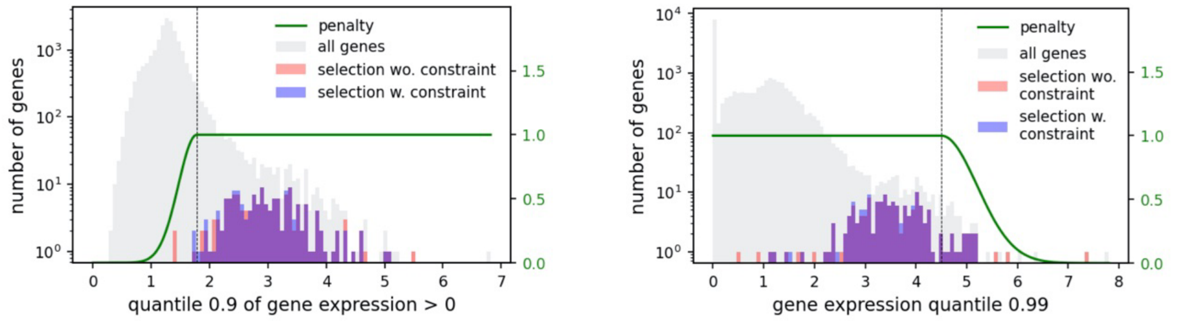

Supplementary Fig. 1: Expression constraint penalties filter out genes beyond expression thresholds. Spapros selections of 150 genes with and without expression constraint penalties. Genes are penalized and not selected as the penalties go to zero.

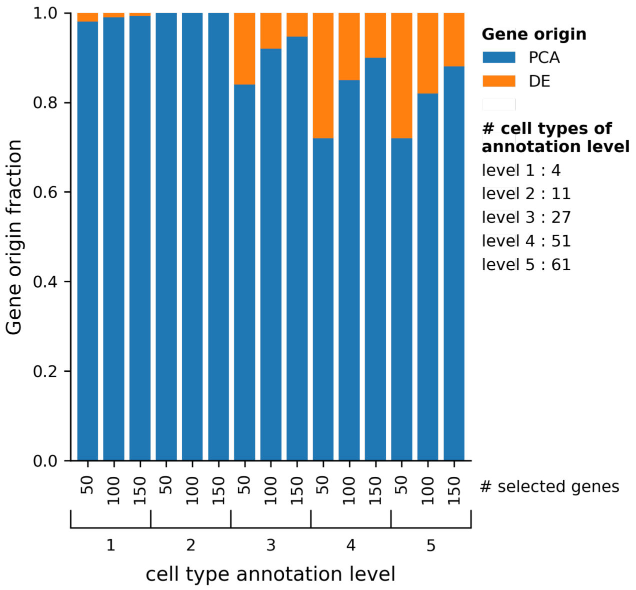

Supplementary Fig. 2: Selection characteristics for different cell type annotation levels. Ratio between selected genes originating from PCA selections or DE trees in Spapros selections. Genes were selected on the human lung cell atlas with different cell type annotation levels.

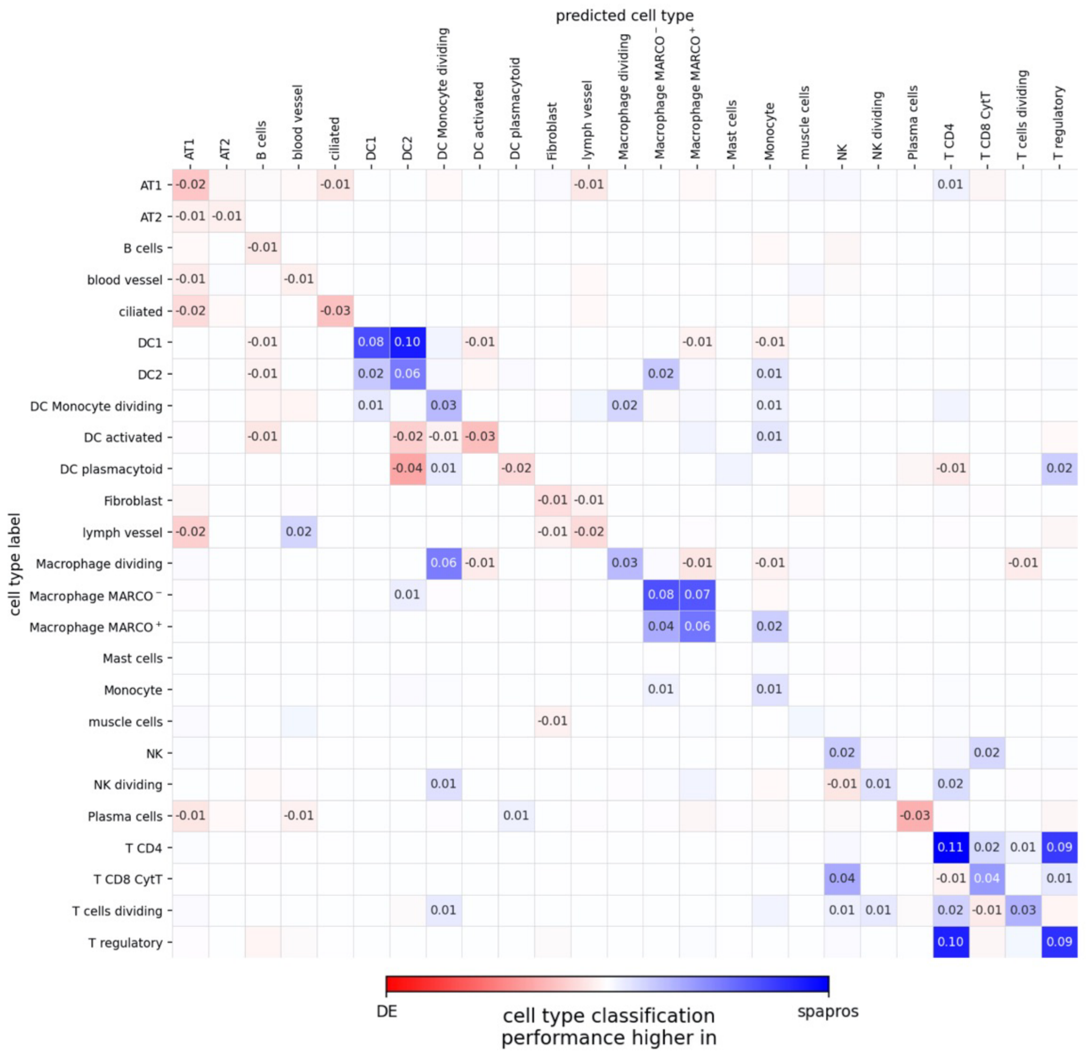

Supplementary Fig. 3: Compare cell type specific classification performance between Spapros and DE. Difference of normalized cell type classification confusion matrices between Spapros and DE selections of gene sets with 50 genes for all cell types in the Madissoon2020 dataset.

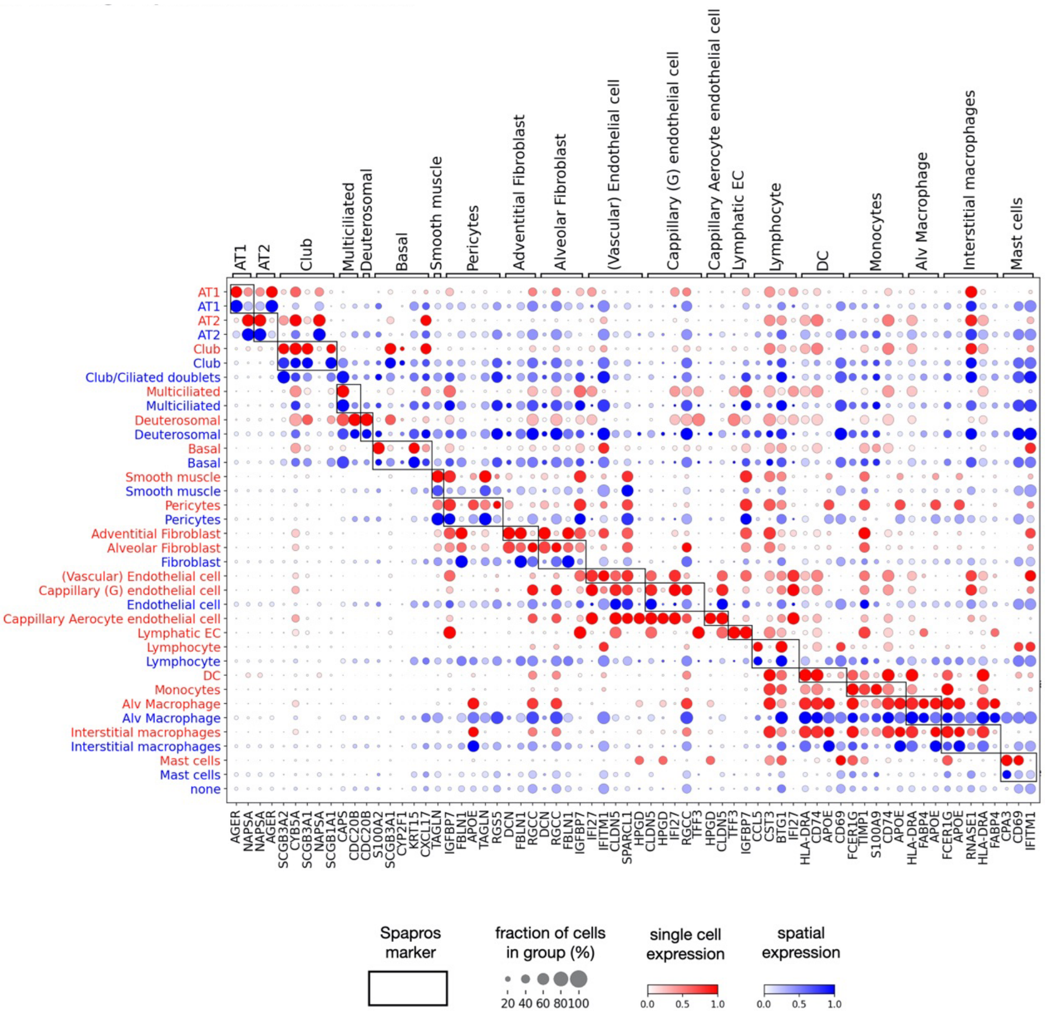

Supplementary Fig. 4: Spatial and scRNA-seq cell type clusters show similar expression profiles. Mean expressions in all spatial cell types of an intralobar lung sample (blue) and in all cell types of the single cell reference (red). The shown genes are identified as the most important genes for cell type identification in the Spapros selection.

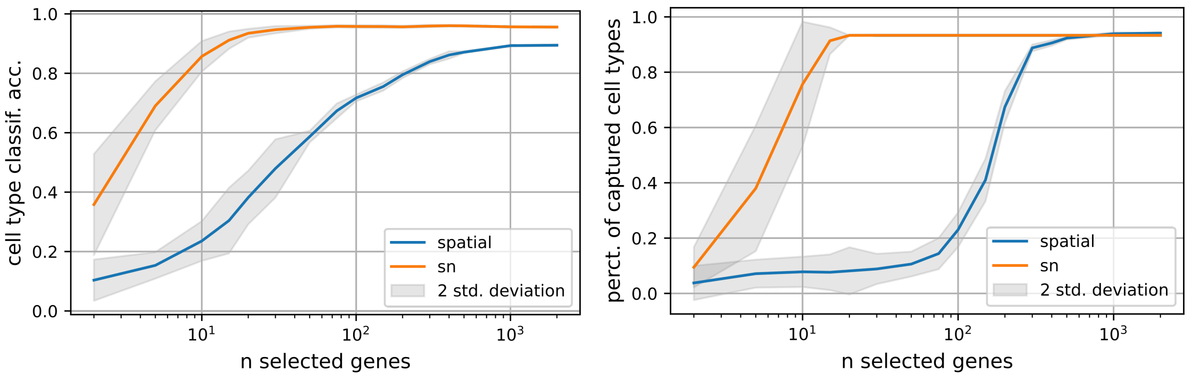

Supplementary Fig. 5: Gene set size dependency of cell type annotation performance. Cell type classification accuracy on snRNA-seq and MERFISH human brain data of Spapros selections on snRNA-seq with different numbers of genes. Data are presented as mean values +/- 2 x SD.

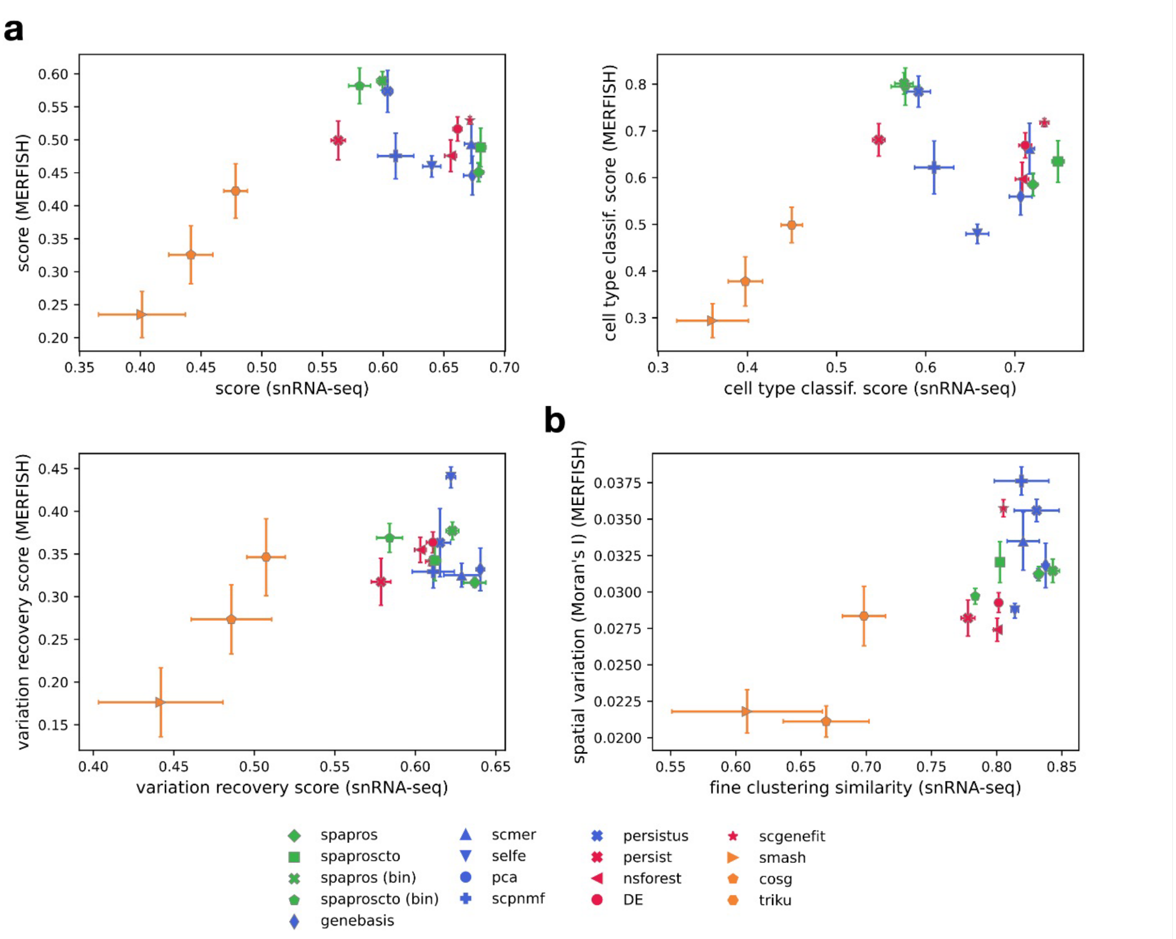

Supplementary Fig. 6: Translation of metrics to spatial data including Spapros on binarised counts. a, Correlation between performance metrics on dissociated and spatial data based on matched snRNA-seq and MERFISH human brain data. Data are presented as mean values +/- SD over selections on 7 bootstrap samples of the snRNA-seq reference for selecting 50 genes. **b,** Correlation between spatial variation metric on the MERFISH data and fine clustering similarity on the snRNA-seq data. Same error bars as in (a).

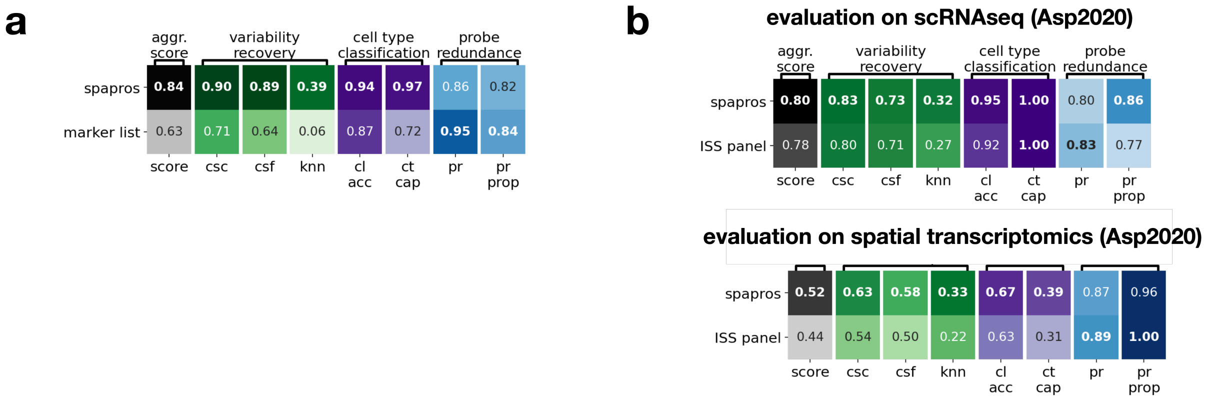

Supplementary Fig. 7: Spapros outperforms classical selection strategies. a, b Performance comparison of probe sets selected with Spapros and (**a**) a curated marker list for lung cell types and (**b**) a gene set used in ISS experiments of Asp 2020 on heart tissue which was based on selections of a single cell dataset and an untargeted spatial transcriptomics data set. Note that the cell type classification metrics on the ST dataset refer to spot based clusters instead of cell type clusters.

Supplementary Note 1

On the Recovery of Spatial Variation with Gene Sets Selected from Dissociated Data

As we lack spatial information in the scRNA-seq reference for gene set selection, our goal is to maximize the likelihood of selecting genes that exhibit spatially varying signals. While not a sufficient criterion for spatial variation, it is necessary that the genes show variation in dissociated data. In a spatial context, such variation could manifest as either locally patterned signals (spatial variation) or randomly distributed variation over space.
Based on our evaluation experiments on matched snRNA-seq and MERFISH data we find the general trend that gene sets optimized for variation recovery on dissociated data show increased capture of spatially relevant variation (Fig. 4e and Extended Data Fig. 2b,c).
Despite the correlation between dissociated and spatial metrics our validation experiment suggests that additional spatial robustness constraints during selections can improve performance on the spatial data. We find that two methods show improved translation of signals to spatial data compared to the expectation from dissociated metrics. Specifically, PERSIST and scPNMF achieve the highest scores in cell type classification and spatial variation on the MERFISH data respectively, despite not being top performers on scRNA-seq (Extended Data Fig. 2c,b). These methods account for the challenges in translation between modalities with specific preprocessing and filtering steps. For PERSIST, this is enabled by optimizing on binarized count data, and for scPNMF, by filtering genes based on correlation with library size and non-unimodality of gene score distributions. Indeed, when applying binarization to Spapros selections, we achieve even slightly higher cell type classification performance on the MERFISH data compared to PERSIST (Supplementary Fig. 4).
Our experiments and recent literature show that spatial robustness constraints are beneficial for current low quality high-plex technologies and less important for other technologies. While the additional robustness constraints enhance translatability to spatial data, it is crucial to consider the extent to which our spatial validation generalizes. The scarcity of high-plex spatial datasets (> 3k genes) limits the generalizability of our validation. Our reliance on a single high-plex MERFISH dataset means our observations are more indicative of trends than definitive rankings. Additionally, and more importantly, recent studies have shown significant variability in the quality of spatial measurements[40,41,75]. These studies suggest that current high-plex solutions (MERFISH, CosMx, STARmap PLUS) exhibit fewer counts per gene and cell, and increased nonspecific signals for individual genes, compared to the higher quality, currently lower-plex methods (MERSCOPE, Xenium). Specifically, as demonstrated with our SCRINSHOT data, individual selected marker genes can effectively recover targeted cell types (Fig. 3a). This was similarly observed in Cook et al.[41], where cell types within delicate spatial structures were recoverable with single marker genes using Xenium, but challenging with high-plex CosMx measurements. Wang et al.[40] also reported similar findings, with more cell types identified across multiple tissues using lower-plex Xenium compared to higher-plex CosMx. These studies provide strong evidence that with the latest advancements in spatial technologies, there is improved translatability between scRNA-seq and spatial data. Taken together, these findings suggest that for panel designs of current high-plex technologies like MERFISH and CosMx, robustness constraints used in methods like PERSIST and scPNMF can be beneficial, while for lower-plex solutions like Xenium and MERSCOPE, better translatability is expected.

Supplemental Note-only reference

75. Hartman, A., & Satija, R., Comparative analysis of multiplexed in situ gene expression profiling technologies, *eLife*, 13 (2024)
