# Supplementary material for: Probe set selection for targeted spatial transcriptomics
Source: Nat Methods. 2024 Nov 18;21(12):2260–70. doi: 10.1038/s41592-024-02496-z (PMC11621025; doi:10.1038/s41592-024-02496-z)
Supplement: Supplementary file 2 — Reporting Summary [file 41592_2024_2496_MOESM2_ESM.pdf]

nature research

Corresponding author(s): Fabian Theis

Last updated by author(s): 24/7/24

Reporting Summary

Nature Research wishes to improve the reproducibility of the work that we publish. This form provides structure for consistency and transparency in reporting. For further information on Nature Research policies, see our Editorial Policies and the Editorial Policy Checklist.

Statistics

For all statistical analyses, confirm that the following items are present in the figure legend, table legend, main text, or Methods section.

n/a	Confirmed	
☐	☒	The exact sample size (*n*) for each experimental group/condition, given as a discrete number and unit of measurement
☐	☒	A statement on whether measurements were taken from distinct samples or whether the same sample was measured repeatedly
☐	☒	The statistical test(s) used AND whether they are one- or two-sided<br>*Only common tests should be described solely by name; describe more complex techniques in the Methods section.*
☒	☐	A description of all covariates tested
☒	☐	A description of any assumptions or corrections, such as tests of normality and adjustment for multiple comparisons
☐	☒	A full description of the statistical parameters including central tendency (e.g. means) or other basic estimates (e.g. regression coefficient) AND variation (e.g. standard deviation) or associated estimates of uncertainty (e.g. confidence intervals)
☐	☒	For null hypothesis testing, the test statistic (e.g. *F*, *t*, *r*) with confidence intervals, effect sizes, degrees of freedom and *P* value noted<br>*Give P values as exact values whenever suitable.*
☒	☐	For Bayesian analysis, information on the choice of priors and Markov chain Monte Carlo settings
☒	☐	For hierarchical and complex designs, identification of the appropriate level for tests and full reporting of outcomes
☐	☒	Estimates of effect sizes (e.g. Cohen's *d*, Pearson's *r*), indicating how they were calculated

Our web collection on statistics for biologists contains articles on many of the points above.

Software and code

Policy information about availability of computer code

Data collection	No software was used.
Data analysis	For the analysis we used our custom python package "spapros" (https://github.com/theislab/spapros) and additional standard python packages. The environment configuration and all used versions are provided in environment.yaml files in https://github.com/theislab/spapros_reproducibility. For the benchmarked external selection methods code or packages in python or R were used. The respective code/package and environments are provided in https://github.com/theislab/spapros_reproducibility. The benchmark was run with our snakemake pipeline https://github.com/theislab/spapros-smk. Spapros' probe design pipeline is given in our separate package https://github.com/HelmholtzAI-Consultants-Munich/oligo-designer-toolsuite. For the analysis of the spatial metrics (CCI score and Moran's I) the packages squidpy (version 1.2.2) and NCEM (0.1.5) were used. For the processing of the SCRINSHOT data the softwares Zen (2.3 Lite), Fiji (ImageJ 1.53c), Cell Profiler (v.3.1.9), and BIAS (version 1.0, available at http://single-cell-technologies.com/download/) were used. For the analysis of the IF samples napari (version 0.4.17) and scikit-image (0.21.0) were used.

For manuscripts utilizing custom algorithms or software that are central to the research but not yet described in published literature, software must be made available to editors and reviewers. We strongly encourage code deposition in a community repository (e.g. GitHub). See the Nature Research guidelines for submitting code & software for further information.

Data

Policy information about availability of data

All manuscripts must include a data availability statement. This statement should provide the following information, where applicable:

- Accession codes, unique identifiers, or web links for publicly available datasets
- A list of figures that have associated raw data
- A description of any restrictions on data availability

The data generated during this study are included in the supplementary information files and on Zenodo at https://doi.org/10.5281/zenodo.10731614. All sc/snRNA-seq, untargeted spatial transcriptomics, and MERFISH datasets are publicly accessible. The datasets used for diverse analyses include Madissoon2020, available at https://www.tissuestabilitycellatlas.org; Krasnow2021 at https://www.synapse.org/#!Synapse:syn21041850; Meyer2022 at https://5locationslung.cellgeni.sanger.ac.uk; Asp2019 ISS at https://doi.org/10.6084/m9.figshare.10058048.v1; Asp2019 single-cell and spatial transcriptomics at https://data.mendeley.com/datasets/mbvhhf8m62/2; Litvinukova2020 at https://www.heartcellatlas.org/v1.html; and HLCA at https://cellxgene.cziscience.com/collections/6f6d381a-7701-4781-935c-db10d30de293. Other datasets used for benchmarking include the Tabula Muris Senis Atlas, accessible via NCBI GEO accession (GSE132042) https://www.ncbi.nlm.nih.gov/geo/query/acc.cgi?acc=GSE132042; the immune cell atlas at https://www.tissueimmunecellatlas.org; liver datasets available via NCBI GEO accession (GSE115469) https://www.ncbi.nlm.nih.gov/geo/query/acc.cgi?acc=GSE115469; thymus data at https://developmental.cellatlas.io/thymus-development; primary motor cortex data (10X_v2, 10X_v3, and SMART samples) available from https://assets.nemoarchive.org/dat-ch1nqb7; PBMC data at https://www.10xgenomics.com/datasets/10-k-pbm-cs-from-a-healthy-donor-v-3-chemistry-3-standard-3-0-0; and bone marrow data from https://figshare.com/projects/Single-cell_proteo-genomic_reference_maps_of_the_human_hematopoietic_system/94469, and GEO accession numbers (GSE201333) https://www.ncbi.nlm.nih.gov/geo/query/acc.cgi?acc=GSE201333, (GSE134355) https://www.ncbi.nlm.nih.gov/geo/query/acc.cgi?acc=GSE134355, and (GSE192616) https://www.ncbi.nlm.nih.gov/geo/query/acc.cgi?acc=GSE192616. The matched brain MERFISH and dissociated data used for the evaluation of spatial metrics can be accessed at https://doi.org/10.5061/dryad.x3ffbg7mw and https://portal.brain-map.org/atlases-and-data/rnaseq/human-mtg-smart-seq, respectively.

Field-specific reporting

Please select the one below that is the best fit for your research. If you are not sure, read the appropriate sections before making your selection.

☒ Life sciences ☐ Behavioural & social sciences ☐ Ecological, evolutionary & environmental sciences

For a reference copy of the document with all sections, see nature.com/documents/nr-reporting-summary-flat.pdf

Life sciences study design

All studies must disclose on these points even when the disclosure is negative.

Sample size	The sample size of each lung region equals 1. As we optimized the selected gene set for cell type recovery and variation recovery over multiple reference samples our method aims to recover major variation common across the population. A single sample that shows that variation in space is therefore representative of the observed cell type patterns and major variation axes in healthy human lungs.
Data exclusions	No data was excluded.
Replication	Our measurements of human lung SCRINSHOT samples with Spapros probesets aimed to show that signals observed in the reference scRNAseq translate to spatial data. In the two measured samples of different lung regions we showed this translatability. Each sample contains several thousand cells and therefore provide a very high effective sample size to show the within sample effects of cell type and intra-cell type variation. Further, our Immunofluorescence labelling on adjacent tissue slides showed within-donor reproducibility of the observed signal. However, with our low sample size regarding number of donors we only show the evidence that these signals do translate to spatial data, we can not make claims about the frequency of the occurrence of the observed signals over patients or show consistent reproducibility of the signal over patient populations.
Randomization	Not relevant to our study as no groups were compared.
Blinding	Not relevant to our study as no groups were compared.

Reporting for specific materials, systems and methods

We require information from authors about some types of materials, experimental systems and methods used in many studies. Here, indicate whether each material, system or method listed is relevant to your study. If you are not sure if a list item applies to your research, read the appropriate section before selecting a response.

Materials & experimental systems

n/a	Involved in the study
☐	☒ Antibodies
☒	☐ Eukaryotic cell lines
☒	☐ Palaeontology and archaeology
☒	☐ Animals and other organisms
☐	☒ Human research participants
☒	☐ Clinical data
☒	☐ Dual use research of concern

Methods

n/a	Involved in the study
☒	☐ ChIP-seq
☒	☐ Flow cytometry
☒	☐ MRI-based neuroimaging

Antibodies

Antibodies used

1. MUC5AC
supplier name: Thermo Fisher Scientific (Invitrogen)
catalog number: MA5-12178
clone name: 45M1
lot number: XC3530341

2. KRT5
supplier name: Biolegend
catalog number: 905901
clone name: Polyclonal, Poly9059
lot number: B29722

3. c-FOS
supplier name: Novus Biologicals
catalog number: NBP1-89065
clone name: Polyclonal
lot number: G119139

Validation

anti-MUC5AC, mouse monoclonal (Thermo Fisher Scientific, MA5-12178). Manufacturer provides information and citation(s) regarding the species-reactivity and usage on tissue sections for immunohistochemistry/immunofluorescence.

anti-Cytokeratin 5, chicken polyclonal (Biolegend, 905901). Manufacturer provides information and citation(s) regarding the species-reactivity and usage on tissue sections for immunohistochemistry/immunofluorescence.

anti-c-FOS, rabbit, polyclonal (Novus Biologicals, NBP1-89065). Manufacturer provides information and citation(s) regarding the species-reactivity and usage on tissue sections for immunohistochemistry/immunofluorescence.

Human research participants

Policy information about studies involving human research participants

Population characteristics

Two samples were obtained from lungs of deceased transplant organ donors. The samples include a biopsy from the distal lung of a 28-year-old male (smoker), and a biopsy from the trachea of a 61-year-old male (non-smoker), both donors having no significant reported lung or tracheal conditions.

Recruitment

Samples were obtained from deceased transplant organ donors by the Cambridge Biorepository for Translational Medicine (CBTM)

Ethics oversight

the NRES Committee of East of England – Cambridge South (15/EE/0152)

Note that full information on the approval of the study protocol must also be provided in the manuscript.
